# Long-Term Effectiveness and Safety of Ustekinumab in Crohn’s Disease: Results from a Large Real-Life Cohort Study

**DOI:** 10.3390/jcm13237192

**Published:** 2024-11-27

**Authors:** Giammarco Mocci, Antonio Tursi, Franco Scaldaferri, Daniele Napolitano, Daniela Pugliese, Ivan Capobianco, Bianca Bartocci, Valentina Blasi, Edoardo V. Savarino, Daria Maniero, Carlo Redavid, Greta Lorenzon, Antonio Cuomo, Laura Donnarumma, Antonietta Gerarda Gravina, Raffaele Pellegrino, Giorgia Bodini, Andrea Pasta, Manuela Marzo, Mariaelena Serio, Antonella Scarcelli, Stefano Rodinò, Ladislava Sebkova, Giovanni Maconi, Giovanni Cataletti, Ileana Luppino, Davide Checchin, Antonio Ferronato, Federica Gaiani, Stefano Kayali, Carla Felice, Giuseppe Pranzo, Domenico Catarella, Dario D’Agostino, Elisabetta Di Bartolo, Giovanni Lombardi, Marta Patturelli, Emanuele Bendia, Laura Bolognini, Daniele Balducci, Claudia Quatraccioni, Francesco Martini, Caterina Mucherino, Elvira D’Antonio, Laura Montesano, Giuliana Vespere, Silvia Sedda, Vittorio D’Onofrio, Leonardo De Luca, Rocco Spagnuolo, Francesco Luzza, Libera Fanigliulo, Giulia Rocco, Carlotta Sacchi, Costantino Zampaletta, Laurino Grossi, Roberto Lorenzetti, Giovanni Aragona, Patrizia Perazzo, Giacomo Forti, Leonardo Allegretta, Alessia Immacolata Cazzato, Stefano Scorza, Fabio Cortellini, Pietro Capone, Guido Daniele Villani, Michela Di Fonzo, Federico Iacopini, Paolo Tonti, Viviana Neve, Raffaele Colucci, Walter Elisei, Rita Monterubbianesi, Roberto Faggiani, Roberta Pica, Cristiano Pagnini, Maria Giovanna Graziani, Maria Carla Di Paolo, Francesca Maria Onidi, Francesco Saba, Maria Pina Dore, Paolo Usai Satta, Marcello Picchio, Alfredo Papa

**Affiliations:** 1 Division of Gastroenterology, AORN “Brotzu” Hospital, 09124 Cagliari, Italy; giammarco.mocci@gmail.com (G.M.); francescamaria.onidi@gmail.com (F.M.O.); fsaba3@gmail.com (F.S.); paolousai@aob.it (P.U.S.); 2 Territorial Gastroenterology Service, ASL BAT, 76123 Andria, Italy; 3 Department of Medical and Surgical Sciences, Catholic University, School of Medicine, 00168 Rome, Italy; 4 Digestive Diseases Centre (CEMAD), Department of Medical and Surgical Sciences, Policlinico Universitario “A. Gemelli” Foundation, IRCCS, 00168 Rome, Italy; franco.scaldaferri@policlinicogemelli.it (F.S.); daniele.napolitano@policlinicogemelli.it (D.N.); daniela.pugliese@policlinicogemelli.it (D.P.); ivan.capobianco01@icatt.it (I.C.); biancab97@hotmail.it (B.B.); v.blasi.97@gmail.com (V.B.); alfredo.papa@unicatt.it (A.P.); 5 School of Medicine, Catholic University, 00168 Rome, Italy; 6 Gastroenterology Unit, Azienda Ospedale-Università di Padova (AOUP), 35100 Padua, Italy; edoardosavarino@gmail.com (E.V.S.); dariamaniero@gmail.com (D.M.); carlo.redavid@studenti.unipd.it (C.R.); gretalorenzon90@gmail.com (G.L.); 7 Division of Gastroenterology, “Umberto I” Hospital, 84014 Nocera Inferiore, Italy; drcuomo@iol.it (A.C.); donnarummalaura@gmail.com (L.D.); 8 Department of Precision Medicine, Hepatogastroenterology and Digestive Endoscopy Unit, University of Campania “Luigi Vanvitelli”, 80138 Naples, Italy; antoniettagerarda.gravina@unicampania.it (A.G.G.); raffaele.pellegrino@unicampania.it (R.P.); 9 Department of Internal Medicine and Medical Specialties, Division of Gastroenterology, IRCCS “San Martino” Hospital, University of Genoa, 86100 Genoa, Italy; bodini.giorgia@gmail.com (G.B.); andreapasta93@gmail.com (A.P.); 10 Division of Gastroenterology, “Veris-Delli Ponti” Hospital, 73020 Scorrano, Italy; manuelamarzo@gmail.com; 11 Division of Gastroenterology, “San Salvatore” Hospital, 61121 Pesaro, Italy; mariaelena.serio@sanita.marche.it (M.S.); antonella.scarcelli@sanita.marche.it (A.S.); 12 Division of Gastroenterology, “Ciaccio-Pugliese” Hospital, 88100 Catanzaro, Italy; srodino@tin.it (S.R.); ladislavasebkova@seznam.cz (L.S.); 13 Gastroenterology Unit, Department Biomedical and Clinical Sciences, “L. Sacco” University Hospital, 20100 Milan, Italy; giovanni.maconi@unimi.it (G.M.); giovanni.cataletti@unimi.it (G.C.); 14 Division of Gastroenterology, “Annunziata” Hospital, 87100 Cosenza, Italy; i.luppino@aocs.it; 15 Division of Gastroenterology, “S Giovanni e Paolo” Hospital, 30100 Mestre−Venezia, Italy; davide.checchin@aulss3.veneto.it; 16 Digestive Endoscopy Unit, ULSS7 Pedemontana, 36014 Santorso, Italy; antonio.ferronato@aulss7.veneto.it; 17 Gastroenterology and Endoscopy Unit, Department of Medicine and Surgery, University of Parma, 43121 Parma, Italy; federica.gaiani@unipr.it (F.G.);; 18 Division of Internal Medicine, “Ca’ Foncello” University Hospital, 31100 Treviso, Italy; carla.felice@aulss2.veneto.it; 19 Ambulatory for IBD Treatment, “Valle D’Itria” Hospital, 74015 Martina Franca, Italy; giuseppepranzo63@gmail.com; 20 Division of Gastroenterology, ARNAS “Garibaldi”, 95100 Catania, Italy; domenicocatarella@gmail.com (D.C.); ddagostino@arnasgaribaldi.it (D.D.); elisabettadiba@gmail.com (E.D.B.); 21 Division of Gastroenterology, AORN “Cardarelli”, 80131 Naples, Italy; giovanni.lombardi@aocardarelli.it (G.L.); martapatturelli@gmail.com (M.P.); 22 Division of Digestive Diseases, Digestive Endoscopy and Inflammatory Bowel Diseases, A.O. “Ospedali Riuniti”, 60121 Ancona, Italy; emanuele.bendia@ospedaliriuniti.marche.it (E.B.); laura.bolognini@ospedaliriuniti.marche.it (L.B.); claudia.quatraccioni@ospedaliriuniti.marche.it (C.Q.); francesco.martini@ospedaliriuniti.marche.it (F.M.); 23 Division of Gastroenterology, Azienda Ospedaliera “S. Anna e S. Sebastiano”, 81100 Caserta, Italy; caterina.mucherino@aorncaserta.it (C.M.); dantonio.elvira@gmail.com (E.D.); la.montesano92@gmail.com (L.M.); 24 Division of Gastroenterology, “Ospedale del Mare”, 80147 Naples, Italy; giuvespere@gmail.com (G.V.); silviasedda@tin.it (S.S.); donofriov@tin.it (V.D.); leonardodeluca@hotmail.com (L.D.L.); 25 Department of Health Science, University of Catanzaro, 88100 Catanzaro, Italy; spagnuolo@unicz.it (R.S.); luzza@unicz.it (F.L.); 26 Division of Gastroenterology, “S.S. Annunziata” Hospital, 74121 Taranto, Italy; liberafani@yahoo.it; 27 Division of Gastroenterology, “Belcolle” Hospital, 01100 Viterbo, Italy; gr.giuliarocco@gmail.com (G.R.); carlotta.sacchi90@gmail.com (C.S.); zcosta@libero.it (C.Z.); 28 Gastroenterology Unit, “Spirito Santo” Hospital, “G d’Annunzio” University, 65121 Pescara, Italy; laurino.grossi@unich.it; 29 Division of Gastroenterology, “Nuovo Regina Margherita” Territorial Hospital, 00153 Rome, Italy; robertolorenzetti58@gmail.com; 30 Division of Gastroenterology, “Guglielmo da Saliceto” Hospital, 29121 Piacenza, Italy; g.aragona@ausl.pc.it (G.A.); p.perazzo@ausl.pc.it (P.P.); 31 Division of Digestive Endoscopy, “S. Maria Goretti” Hospital, 04100 Latina, Italy; giacomo.forti@ausl.latina.it; 32 Division of Gastroenterology, “Santa Caterina Novella” Hospital, 73013 Galatina, Italy; leonardo.allegretta@tin.it (L.A.); alessiacazzato@gmail.com (A.I.C.); stefano.scorza@gmail.com (S.S.); 33 Division of Gastroenterology, “Infermi” Hospital, 47921 Rimini, Italy; fabio.cortellini@auslromagna.it; 34 Division of Gastroenterology, “T. Maresca” Hospital, 80059 Torre del Greco, Italy; pietrocapone@hotmail.com (P.C.); guidodaniele.villani@ospedalideicolli.it (G.D.V.); 35 Division of Gastroenterology, “Ospedale dei Castelli”, 00040 Ariccia, Italy; micheladifonzo@gmail.com (M.D.F.); federico.iacopini@aslroma6.it (F.I.); 36 Division of Gastroenterology, “A. Perrino” Hospital, 72100 Brindisi, Italy; paolo.tonti@libero.it (P.T.); viviana.neve@libero.it (V.N.); 37 Digestive Endoscopy Unit, “San Matteo degli Infermi” Hospital, 06049 Spoleto, Italy; colucciraffaele@tiscali.it; 38 Division of Gastroenterology, A.O. “S. Camillo-Folanini”, 00152 Rome, Italy; walter_elisei@hotmail.com (W.E.); rita.monterubbianesi@gmail.com (R.M.); faggiani.r@tin.it (R.F.); 39 Division of Gastroenterology, IBD Unit, “S. Pertini” Hospital, 00157 Rome, Italy; robertapica5@gmail.com; 40 Division of Gastroenterology, “S. Giovanni-Addolorata” Hospital, 00184 Rome, Italy; cristianopagnini@gmail.com (C.P.); mg.graziani@virgilio.it (M.G.G.); mcdipaolo@hsangiovanni.roma.it (M.C.D.P.); 41 Department of Medicine, Surgery and Pharmacy, University of Sassari, 07100 Sassari, Italy; mpdore@uniss.it; 42 Division of General Surgery, “P. Colombo” Hospital, ASL Roma 6, 00049 Velletri, Italy; marcellopicchio2@gmail.com

**Keywords:** Crohn’s disease, ustekinumab, remission, response, re-induction, safety

## Abstract

**Background:** Ustekinumab (UST) is an interleukin-12/interleukin-23 receptor antagonist approved for the treatment of Crohn’s disease (CD). Only limited real-life data on the long-term outcomes of CD patients treated with UST are available. This study assessed UST’s long-term effectiveness and safety in a large population-based cohort of moderate to severe CD patients. **Methods:** This was a multicenter, retrospective, observational cohort study that included both naïve and biologic-experienced patients treated with UST who achieved clinical remission or clinical response after at least one year of treatment. Clinical activity was scored according to the Harvey–Bradshaw Index (HBI). The primary endpoints were the maintenance or achievement of clinical remission after a further 12-month period of treatment, defined as an HBI of ≤5, and safety. Other endpoints included steroid-free remission, mucosal healing (MH), steroid discontinuation, and the need for treatment optimization during the follow-up. **Results:** Out of 562 CD patients, after an overall 24-month follow-up, clinical remission was present in 450 (80.0%) patients, and at 12 months, clinical remission was observed in 417/437 (95.4%) patients; 33/125 (26.4%) showed clinical response at 12 months (*p* = 0.000). A total of 38/103 (36.9%) patients achieved MH. Only 2.1% (12/562), 3% (17/562), and 1.1% (6/562) of patients required surgery, optimization, and re-induction, respectively. Adverse events occurred in eight patients (1.42%). According to a multivariate analysis, the only predictor of long-term remission was the presence of remission at the 12-month follow-up (*p* = 0.000). **Conclusions**: Long-term treatment with UST presents good efficacy and safety profiles in CD patients, especially for patients who achieve remission after one year.

## 1. Introduction

Crohn’s disease (CD) is a chronic, transmural, granulomatous inflammatory condition of the gastrointestinal (GI) tract of unknown etiology [1]. CD’s global incidence and prevalence have increased along with an improved understanding of the disease’s clinical presentation, diagnosis, and natural history [2,3,4]. A relapsing and remitting progression characterizes the clinical course of the disease, and an aggressive therapeutic approach is often required to prevent complications from occurring [5].

Following the discovery of the critical pathogenetic role of tumor necrosis factor-α (TNF-α) in inflammatory bowel disease (IBD), monoclonal anti-TNFα antibodies have been developed and successfully adopted in clinical practice [5]. However, many patients do not respond to anti-TNF treatment or experience a secondary loss of response or intolerance to treatment due to intolerance, immunogenicity, or mechanistic failure [6,7]. Furthermore, there is a risk of infectious complications attributable to non-specific TNF-mediated inhibition [8,9]. Thus, novel therapeutic agents targeting alternative pathogenetic pathways have been investigated and approved for IBD treatment.

Ustekinumab (UST) is a monoclonal antibody blocking the p40 subunit of the interleukin (IL) 12/23 [10] that was granted marketing authorization in November 2016 by the European Medicines Agency for the treatment of moderate-to-severe CD in adult patients with inadequate response, loss of response, or intolerance to either conventional therapies or biologics [11]. It is also currently approved for the treatment of psoriasis and ulcerative colitis [12,13]

The efficacy and safety of UST in CD over a one-year period has been previously established in UNITI-1 and UNITI-2 (8 weeks) and IM-UNITI (44 weeks) controlled studies [14,15]. In the 3-year extension of this trial, 38.0% of UST induction responders receiving the drug every 12 weeks, and 43.0% receiving the drug every eight weeks, were in remission at week 152 [16]. Finally, 34.4% of patients in the every-8-weeks group and 28.7% in the every-12-weeks group were in clinical remission at week 252 [17]. 

Several real-world cohort studies have assessed the effectiveness and safety outcomes up to 52 weeks, confirming its efficacy in daily practice [18,19,20,21,22,23,24,25,26,27,28,29,30,31,32,33,34,35]. However, long-term outcomes beyond 52 weeks have only been assessed in a few real-world studies, often constrained by small numbers of enrolled patients or other limitations such as monocentric enrollment or the lack of specific assessment [36,37,38,39,40,41,42,43,44].

The present study aimed to assess UST’s long-term effectiveness and safety in a large cohort of adult patients with CD with a minimum follow-up time of twelve months. We also set out to identify clinical and laboratory parameters that may influence the response to UST in the long term.

## 2. Methods

We conducted a retrospective, observational, multicenter study that included both naïve and biologic-experienced CD outpatients (including those who experienced failed treatments with anti-TNFa antibodies and vedolizumab) treated with UST in 40 Italian IBD centers who were in clinical remission or showed clinical response after completing at least one year of treatment.

Men and women at least 18 years of age with a CD diagnosis established according to standard endoscopic and/or radiologic and/or histological criteria were considered eligible [45]. Exclusion criteria included patients presenting with a diagnosis of unclassified IBD, intestinal strictures or complications accompanied by surgical indications, a stoma, extensive bowel resection (≥2 bowel surgical resection and/or 50 cm of ileal resection) [46], intestinal failure, and those receiving dual biological therapy (i.e., simultaneously using UST plus another biologic agent or a small molecule drug).

A common database was created to collect demographic and clinical data. At baseline, we collected the following data: gender, age at diagnosis, current smoking status, presence of comorbidities, previous appendectomy, previous surgery for CD, the extension of the disease according to the Montreal classification, disease duration, previous immunosuppressive and biologic therapies (anti-TNFα and/or anti-integrin), concomitant medications, fecal calprotectin (FC), C-reactive protein (PCR), erythrocyte sedimentation rate (ESR), Harvey–Bradshaw Index (HBI), Simple Endoscopic Score for CD (SES-CD), and Rutgeerts score for endoscopy (for patients with previous surgery).

We conducted the study according to the clinical practice guidelines and following the principles of the Declaration of Helsinki. All patients provided written informed consent before undergoing endoscopy and UST treatment. The reference center (Brotzu Hospital, Cagliari, Italy, PROT. PG/2020/9414, 29 April 2020) obtained ethics committee approval for this retrospective study, and this approval was accepted by the other centers.

### 2.1. Study Treatment

All patients were treated uniformly during the induction phase with a baseline intravenous infusion adapted to the following weight ranges: <55 kg: 260 mg; 55–85 kg: 390 mg; >85 kg: 520 mg. After induction, subcutaneous UST 90 mg was administered every eight weeks to maintain remission.

This interval of administration for maintenance treatment was chosen by all the investigators, considering that most of the patients had experienced the failure of one or more biological agents.

The investigators were left to judge the need for treatment discontinuation or dose escalation during the every-4-week therapy. They were also left to judge concomitant medications, such as oral and topical aminosalicylates, steroids, and/or immunosuppressants.

### 2.2. Clinical Assessment at Baseline and During the Follow-Up

The Montreal classification [47] was used to assess disease extension, while the Harvey–Bradshaw Index (HBI) [48] score was used to evaluate the activity of the disease. All the patients included in the study expressed active disease at the time of UST enrollment, defined as an HBI score > 5 points [48], despite concomitant treatment. Patients were clinically evaluated at entry and then again after 2, 6, 12, 18, and 24 months and subsequently, every 12 months, or in the case of loss of clinical response. CRP and FC levels were obtained at baseline and then at 2, 6, 12, 18, 24 and after that, every twelve months or in case of loss of clinical response.

### 2.3. Endoscopy Assessment at Baseline and During the Follow-Up

All patients underwent a colonoscopy before starting UST treatment, per standard protocol, in the participating centers. After 1-year of follow-up or earlier, as well as later in the study, depending on the patient’s clinical history and the clinician’s discretion, an ileocolonoscopy, with biopsies, was offered to monitor disease activity or for cancer surveillance. The Simple Endoscopic Score for CD (SES-CD) and Rutgeerts score (for patients with prior surgery) were used to assess endoscopic severity [49,50,51]. Central reading for the assessment of the endoscopic activity was not performed.

### 2.4. Outcomes

The primary outcome was to assess the effectiveness of UST in terms of the maintenance or achieving clinical remission in CD patients who, after 12 months of treatment with UST, were in clinical remission (HBI of ≤5) or presented a clinical response, with a mild clinical activity (HBI 6–8), respectively. The co-primary outcome was the safety of UST, defined as the absence of adverse events (AEs) during the follow-up. The AEs were subdivided into early events (during the infusions) and late events (at least one week after the infusion/injection) and graded as mild (which did not require treatment interruption) and severe (which instead required interruption of the treatment) [52].

In addition, this study provided several secondary outcomes:

- Mucosal healing, defined as SES-CD ≤ 2 in CD patients;
- Reduction of steroid use during the study (defined as the use of systemic or topic steroids);
- Maintenance of steroid-free remission during the study;
- Occurrence of any surgical procedure related to the disease in CD;
- UST optimization, defined as the reduction of the time between the injections from eight to four weeks) during follow-up;
- CRP, FC, and HBI variations during follow-up;
- Re-induction of remission, defined as re-induction with intravenous infusion of either ustekinumab 260, 390, or 520 mg, according to the weight per prescribing guidelines [53].

### 2.5. Statistical Analysis

The data are presented using descriptive statistics. Continuous variables are expressed as the median and interquartile range (IQR); dichotomous or ordinal variables are presented as the number (percentage) of patients.

Clinical remission was considered as the primary endpoint. The predictive value of the clinical parameters was evaluated using time-to-event methods for censored observations because of the varying length of follow-up. Follow-up times were calculated from the date of diagnosis to the date of event or censorship. The time-to-event analysis used Kaplan–Meier estimates to draw the cumulative incidence curves, which were compared using log-rank tests and univariate and multivariate Cox proportional hazards models of the prognostic variables. The hazard ratios are presented with the 95% confidence intervals and *p*-values. A ratio higher than unity implies that an event has a higher probability than that of the reference group. The Friedman test was used to investigate any change in CRP and FC levels during follow-up. *p*-values < 0.05 were considered to be statistically significant.

## 3. Results

### 3.1. Baseline Characteristics (At 12 Months After Beginning UST Treatment)

A total of 562 were included; the median follow-up was 12 (18–36) months. Table 1 shows the baseline characteristics of patients at one year of follow-up. CD was located in the ileal and ileocolonic tracts in most patients, and stricturing disease was the most common phenotype (47.5%). Of note, approximately one patient out of four was a current smoker, and more than half (55.5%) had undergone intestinal resections in the past.

Regarding concomitant therapies, most of the patients were taking mesalazine, whereas only 15.8% were on steroids.

Furthermore, a relevant proportion of patients experienced previous failure with one or more (86.9%) lines of therapy using anti-TNFs and/or anti-integrin.

Concerning disease activity, the median CRP was 3 (1–5) mg/dL and FC 148 (80–244) μg/g, while the median HBI was 4 (2–5), and the SES-CD was 5 (2–8).

At one year of follow-up, 437 (77.8%) patients had achieved clinical remission, while 125 (22.2%) had shown a clinical response and were still displaying mild clinical activity.

### 3.2. Primary Outcomes

**Figure 1:**
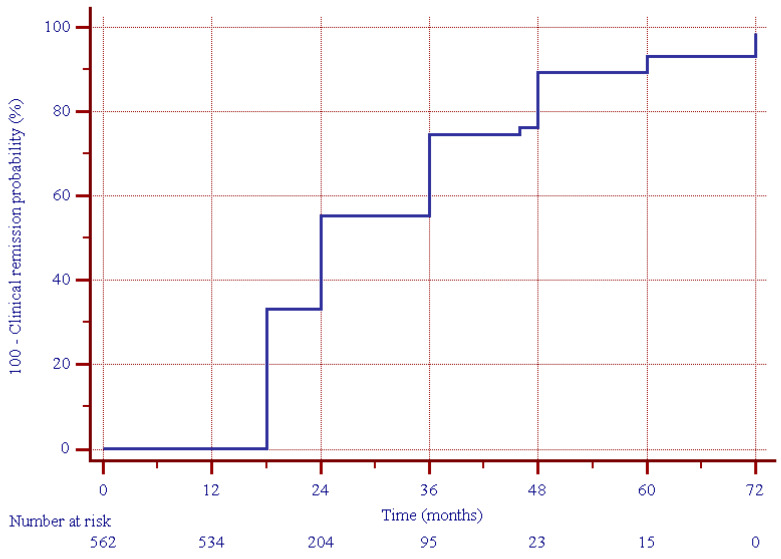
Estimated cumulative clinical remission probability during follow-up in the study group.

**Figure 2:**
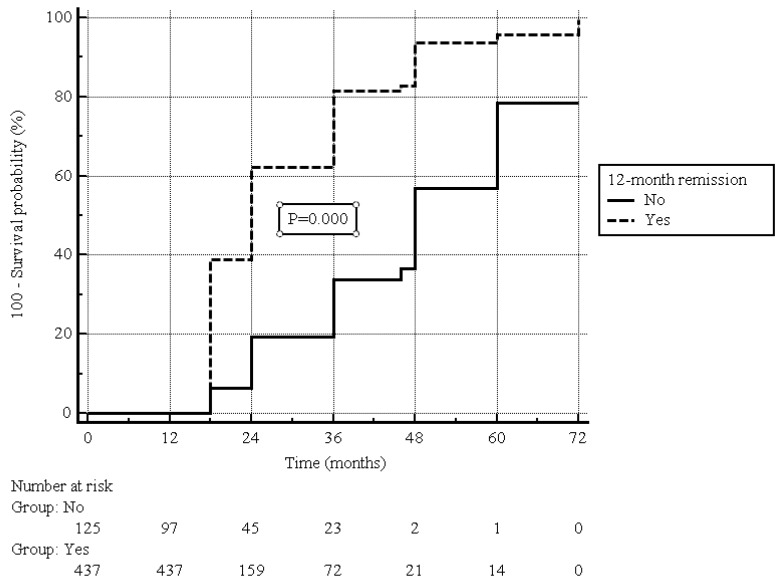
Estimated cumulative clinical remission probability during follow-up in patients with or without clinical remission at 12-month follow-up; log-rank test.

After a median (IQR) of 12 (6–24) months from the enrollment (i.e., two years after the beginning of UST treatment), clinical remission was present in 450 (80.0%) patients (Figure 1), including 417 out of 437 (95.4%) patients who were in clinical remission at the 12-month follow-up, and 33 out of 125 (26.4%) patients showing clinical response at the 12-month follow-up (*p* = 0.000, Figure 2).

Optimization was performed in 17 (3.0%) patients (13 patients with clinical remission and 4 patients without clinical remission) and re-induction in 6 patients (5 patients with clinical remission and 1 patient without clinical remission).

According to multivariate analysis, it was determined that the only predictor of long-term remission was the presence of remission at the 12-month follow-up (*p* = 0.000; Table 2). In the 33 patients with a clinical response at enrollment, long-term clinical remission was achieved in 29 (87.9) non-smokers and 4 (12.9) active smokers (*p* = 0.030).

### 3.3. Secondary Outcomes

**Figure 3:**
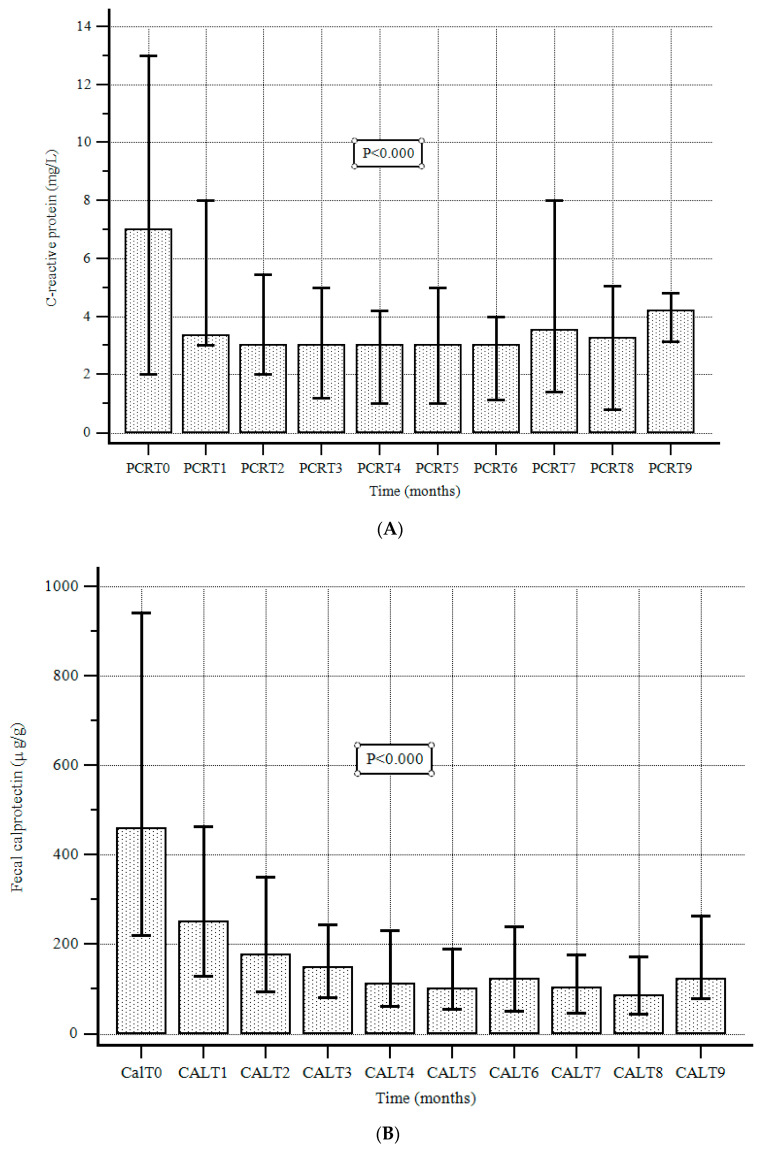
(**A**). C-reactive protein values at baseline and during follow-up. Data are expressed as the median and interquartile range (error bars), according to the Friedman test. (**B**). Fecal calprotectin values at baseline and during follow-up. Data are expressed as the median and interquartile range (error bars), according to the Friedman test.

At the beginning of the treatment with UST, median FC and CRP values were 459 µg/g (220–942) and 7 mg/L (2–13), respectively. During the follow-up, the C-reactive protein and FC values were significantly reduced compared to the baseline and 1-year values (Figure 3A,B).

The median (IQR) CRP level at the end of the follow-up was 4.4 mg/L (3.2–4.8), with a significant reduction compared to baseline values (*p* < 0.000). Moreover, the median (IQR) FC level was 85 μg/g (42.5–172.0), with a significant reduction compared to baseline values (*p* < 0.000).

Steroids were maintained in only 22 (4.9%) patients exhibiting clinical remission and 21 (18.8%) patients showing clinical response (*p* = 0.000).

An endoscopic assessment was performed in 103 patients 12 (6–24) months after enrollment (1 year after the beginning of UST treatment), and mucosal healing was achieved in 38 (36.9%) patients.

Overall, 17 (3%) patients requested dosage optimization, whereas 6 (1.1%) patients chose re-induction.

Finally, surgery occurred in 12 (2.1%) patients after a median of 12 (6–24) months from the enrollment (1 year after the beginning of UST treatment) (Table 2).

### 3.4. Safety Profile

Table 3 reports adverse events (AEs). During follow-up, AEs occurred in 8 (1.42%) patients.

Adverse events were all mild to moderate, except for one tracheal stenosis that required treatment discontinuation. We recorded only one case of simplex herpes zoster (a single dermatome was involved, and the infection resolved within four weeks). This patient was not vaccinated against herpes zoster, as the large majority (>95%) of the population was enrolled before the recombinant vaccine became available in Italy in March 2021.

No differences were found between the patients with or without clinical remission at baseline.

## 4. Discussion

A growing body of evidence from RW data for UST provides credible evidence for its effectiveness and safety for treating moderately to severely active CD. Since the publication of the UNITI pivotal trials, several real-life studies from Europe, Asia, and North and South America have been conducted, confirming its efficacy in daily practice [14,15,16,18,19,20,21,22,23,24,25,26,27,28,29,30,31,32,33,34,35]. However, long-term data reflecting their use in real-life clinical practice are still being compiled [36,37,38,39,40,41,42,43,44]. To our knowledge, this multicenter real-world study is the largest assessing the effectiveness and safety of UST in a real-life long-term scenario. We showed that most patients reaching remission after one year of treatment can maintain remission for 12 months. Moreover, we found that about one-fourth of patients showing clinical response after one year of treatment can reach remission even after 12 months of treatment, and this late remission is easier for no-smoking patients to achieve.

It is essential to highlight the characteristics of our population at baseline to understand and interpret the results of our study. First, in a cohort of 562 patients, most of whom had already been treated with other biologics, 77% were in clinical remission at baseline. These results appear to be better not only than those of the pivotal studies UNITI-1 and UNITI-2 [14,15] but also than those from a recent meta-analysis of real-life studies, in which a pooled remission rate of 40% was observed at 52 weeks [54]. A possible explanation could be the high proportion of patients who started UST to prevent post-operative recurrence (POR) rather than for the treatment of active CD.

Second, the primary endpoint, clinical remission over 12 months, was achieved in 80% of patients with a median follow-up of 24 months. Again, compared with the literature data, our results seem more favorable. The IM-UNITI trial found that 38.0% of UST induction responders receiving the drug every 12 weeks and 43.0% receiving the drug every eight weeks were in remission at week 152 [16]. Finally, 34.4% of patients in the every-8-weeks group and 28.7% in the every-12-weeks group were in clinical remission at week 252 [17]. Of course, our results could be explained by the 8-week regimen generally adopted in real life in Italy rather than, as previously reported, to the large proportion of patients treated for the prevention of POR [28,33].

The most robust data of our study are related to treatment persistence. Almost all (95%) of patients in remission 12 months after starting treatment maintained remission for at least another 12 months. In addition, a significant proportion (approximatively 25%) of “late remitters”, i.e., patients not in remission at baseline, achieve remission during follow-up. This finding is relevant in practice and should encourage clinicians to continue maintenance therapy for 12 months. On the other hand, another therapeutic option should be considered in the absence of clinical signs of efficacy and with objective markers of disease activity.

We also analyzed factors that might predict long-term clinical remission with UST. We found that remission at the 12-month follow-up was the only predictor of long-term remission using multivariate analysis. Interestingly, confirming a finding already reported in our previous research regarding treating CD patients with UST [33], clinical remission seems to be independent of the number of different biological agents previously used, and this also applies to the subgroup of patients who achieve remission during follow-up. Overall, these data lead to two important considerations. The first is that UST is effective in obtaining remission even in patients already treated with more than one monoclonal antibody. This is confirmed by the very low number of patients requiring dose optimization or reinduction to reach or maintain remission (4.1%). This rate is too low to allow for an adequate sub-analysis, even if the recent POWER study identifies some parameters that are more likely to achieve a clinical response after reinduction [55]. The second is that UST may work better in CD patients after the first treatment with an anti-TNFα. In particular, with a view toward the rational sequencing of biological drugs, the evidence that non-responders to anti-TNF therapy show an increase in apoptosis-resistant, IL23-positive T cells, which promote inflammation, makes the IL 12/23 and IL 23 blockage particularly attractive [56].

Concerning the secondary end-points, we found that UST also exhibits a significant efficacy in reaching other important clinical outcomes. Both CRP and CF significantly dropped under treatment with UST, and MH was present in almost 40% of patients. This confirms that the clinical remission derived from UST is closely related to MH, even if the small number of endoscopic control patients limits these results. During follow-up, there was a reduction in steroid use, with more than 95% of patients in clinical remission also being steroid-free. Finally, overall, dosage optimization was requested in 17 (3%) patients, whereas re-induction was used in 6 (1.1%).

The safety profile of UST is very favorable according to pivotal trials and the IM-UNITI trial [14,15,16]. This favorable profile has also been confirmed in real life. In CD, the mean rate of the AEs is about 11%, with the large majority of them being mild and not requiring the discontinuation of treatment. This study confirms an excellent safety profile, since AEs occurred in only 8 (1.42%) patients.

Adverse events were mild to moderate, including only one case of simplex herpes zoster in an unvaccinated patient. There was only one case of serious AEs, a tracheal stenosis that required discontinuation of treatment.

This study has both strengths and limitations. The main strengths lie in the large number of patients enrolled, the reasonably good length of follow-up, and the use of clinical scores to evaluate the disease. An adjunctive strength is its long-term evaluation of drug safety. Finally, for the first time, we found that one-fourth of patients showing clinical response after one year of treatment can reach remission even after 12 months of treatment, and this late remission is easier for non-smoking patients to achieve. This means that in patients with a clinical response at one year, it seems appropriate to continue the therapy beyond one year since, especially for non-smokers [57], the chances of achieving delayed remission are significant.

The primary limitations lie in the retrospective nature of the study, which does not permit the enrollment of patients at the same time through the follow-up (for both clinical and endoscopic follow-up). The second limitation is that we mainly enrolled outpatients with less aggressive, or generally moderate rather than severe, disease behavior. This could explain the superior results of this study compared to those for the UNITI trial [17]. The third limitation is that, as this was a multicentric study involving 40 centers, it was impossible to guarantee that each patient received standardized management. For example, there is no clear indication of when to perform endoscopic control, which is generally reserved for patients who do not respond to the treatment, in real-life patients under treatment with biologics [58]. This could explain the lower rate (about 20%) of patients undergoing endoscopic follow-up recorded in this study.

## 5. Conclusions

The results of our real-world, multicenter study found that UST is effective and safe in managing outpatient CD during long-term follow-up. Interestingly, we identified some parameters that can help the physician predict the long-term efficacy of this drug. Further studies featuring large sample sizes and prospective designs are needed to confirm these findings.

## Figures and Tables

**Table 1:** Characteristics of the study group.

Male Sex	312 (55.5)
Median (IQR) age at diagnosis, years	45 (32–57)
Current smokers	150 (26.7)
Previous appendectomy	141 (25.1)
Previous surgery for CD	312 (55.5)
Montreal classification	
Age at diagnosis (years)	
17–39	225 (40.0)
≥40	337 (60.0)
Location	
Isolated ileal disease	199 (35.4)
Isolated colonic disease	81 (14.4)
Ileocolonic disease	282 (50.2)
Concomitant perianal disease	71 (12.6)
Behavior	
Non stricturing, non-penetrating	205 (36.5)
Stricturing	267 (47.5)
Penetrating	90 (16.0)
Median (IQR) disease duration, years	11 (7–19)
Failure of other biologics Naïve	488 (86.8) 74 (13.2)
Steroid-free	519 (92.3)
Concomitant medications	
Mesalazine	316 (56.2)
Azathioprine Median (IQR) fecal calprotectin (µg/g)	21 (3.7) 148 (80–244)
Median (IQR) CRP (mg/L)	3 (1–5)
Median (IRQ) HBI	4 (2–5)
Median (IRQ) SES-CD (130 pts)	5 (2–8)
Rutgeerts score (110 pts)	1 (1–2)
Clinical response	125 (22.2)
Clinical remission	437 (77.8)

Data are given as the number (percentage) of patients, unless otherwise indicated. IQR, interquartile range; CRP, C-reactive protein; HBI, Harvey–Bradshaw Index; SES-CD, Simple Endoscopic Score for Crohn’s disease.

**Table 2:** Predictors of long-term clinical remission.

	Total	Remission	No Remission	Univariate Analysis				Multivariate Analysis		
Variable	562	450 (80.1)	112 (19.9)	HR	95% CI	*p*		HR	95% CI	*p*
Sex										
Female	250 (44.5)	198 (79.2)	52 (20.8)				Ref.			
Male	312 (55.5)	252 (80.8)	60 (19.2)	0.99	0.83–1.20	0.954		0.96	0.80–1.16	0.695
Current smokers										
No	412 (73.3)	332 (80.6)	80 (19.4)							
Yes	150 (26.7)	118 (78.7)	32 (21.3)	0.88	0.72–1.08	0.131		0.87	0.70–1.08	0.201
Previous surgery for CD										
No	250 (44.5)	201 (44.7)	49 (43.7)				Ref.			
Yes	312 (55.5)	249 (55.3)	63 (56.2)	0.98	0.82–1.18	0.860		1.05	0.87–1.27	0.622
Previous appendectomy										
No	421 (74.9)	348 (82.7)	73 (17.3)				Ref.			
Yes	141 (25.1)	102 (72.3)	39 (27.7)	0.94	0.76–1.17	0.499		0.90	0.85–1.46	0.087
Age										
18–39	225 (40.0)	185 (82.2)	40 (17.8)				Ref.			
≥40	337 (60.0)	256 (78.6)	72 (21.4)	0.88	0.73–1.07	0.100		0.86	0.71–1.04	0.129
Location										
Other	280 (49.8)	218 (77.9)	62 (22.1)				Ref.			
Ileocolonic	282 (50.2)	232 (82.3)	50 (17.7)	1.06	0.88–1.28	0.401		1.03	0.86–1.25	0.712
Behavior										
Non stricturing, non-penetrating	205 (36.5)	167 (81.5)	38 (18.5)				Ref.			
Stricturing/penetrating	357 (63.5)	283 (79.3)	74 (20.7)	0.92	0.75–1.11	0.258		0.91	0.75–1.11	0.362
Naïve to biologics										
No	488 (86.8)	397 (88.2)	91 (81,2)				Ref.			
Yes	74 (13.2)	53 (11.8)	21 (18.8)	0.98	0.73–1.31	0.873		0.94	0.71–1.401	0.241
Non-response to biologics										
No	229 (40.7)	183 (79.9)	46 (20.1)				Ref.			
Yes	333 (59.3)	267 (80.2)	66 (19.8)	1.15	0.95–1.38	0.067		1.27	1.03–1.56	0.028
Clinical response										
No	62 (11.0)	15 (3.3)	47 (42.0)				Ref.			
Yes	500 (89.0)	435 (96.7)	65 (58.0)	3.55	2.64–4.78	0.000		1.44	0.725–2.88	0.295
Clinical remission										
No	125 (22.2)	33 (7.3)	92 (82.1)				Ref.			
Yes	437 (77.8)	417 (92.7)	20 (17.9)	3.15	2.50–3.97	0.000		2.95	1.82–4.78	0.000

Data are given as the number (percentage) of patients, including HR, hazard ratio, and CI, confidence interval.

**Table 3:** Adverse events.

	Group A (437/562)	Group B (125/562)	*p*-Value
Total Adverse Events (AE)	6 (1.4%)	2 (1.6%)	ns
Mild-Moderate AE - Allergy - Erythema nodosum - Herpes zoster - Dermatitis - Urinary tract Infection - Orchitis	1 (0.2%) 1 (0.2%) 1 (0.2%) 1 (0.2%) - -	- - - - 1 (0.8%) 1 (0.8%)	ns ns ns ns ns ns
Severe AE - Tracheal stenosis	1 (0.2%)	-	ns

ns: not significant.

## Data Availability

Data from this study are available from the corresponding authors upon reasonable request.

## References

[1] Baumgart D.C., Sandborn W.J. (2012). Crohn’s disease. Lancet.

[2] Molodecky N.A., Soon I.S., Rabi D.M., Ghali W.A., Ferris M., Chernoff G., Benchimol E.I., Panaccione R., Ghosh S., Barkema H.W. (2012). Based on systematic review, increasing incidence and prevalence of the inflammatory bowel diseases with time. Gastroenterology.

[3] Jones G.R., Lyons M., Plevris N., Jenkinson P.W., Bisset C., Burgess C., Din S., Fulforth J., Henderson P., Ho G.T. (2019). IBD prevalence in Lothian, Scotland, derived by capture-recapture methodology. Gut.

[4] Kaplan G.G. (2015). The global burden of IBD: From 2015 to 2025. Nat. Rev. Gastroenterol. Hepatol..

[5] Torres J., Mehandru S., Colombel J.F., Peyrin-Biroulet L. (2017). Crohn’s disease. Lancet.

[6] Kennedy N.A., Heap G.A., Green H.D., Hamilton B., Bewshea C., Walker G.J., Thomas A., Nice R., Perry M.H., Bouri S. (2019). Predictors of anti-TNF treatment failure in anti-TNF-naive patients with active luminal Crohn’s disease: A prospective, multicentre, cohort study. Lancet Gastroenterol. Hepatol..

[7] Baert F., Noman M., Vermeire S., Van Assche G., D’Haens G., Carbonez A., Rutgeerts P. (2003). Influence of immunogenicity on the long-term efficacy of infliximab in Crohn’s disease. N. Engl. J. Med..

[8] Ford A.C., Sandborn W.J., Khan K.J., Hanauer S.B., Talley N.J., Moayyedi P. (2011). Efficacy of biological therapies in inflammatory bowel disease: Systematic review and meta-analysis. Am. J. Gastroenterol..

[9] Bonovas S., Fiorino G., Allocca M., Lytras T., Nikolopoulos G.K., Peyrin-Biroulet L., Danese S. (2016). Biologic therapies and risk of infection and malignancy in patients with inflammatory bowel disease: A systematic review and network meta-analysis. Clin. Gastroenterol. Hepatol..

[10] Teng M.W., Bowman E.P., McElwee J.J., Smyth M.J., Casanova J.-L., Cooper A.M., Cua D.J. (2015). IL-12 and IL-23 cytokines: From discovery to targeted therapies for immune-mediated inflammatory diseases. Nat. Med..

[11] Stelara EMA Product Information. http://ec.europa.eu/health/documents/community-register/html/h494.htm.

[12] Zaghi D., Krueger G.G., Callis Duffin K. (2012). Ustekinumab: A review in the treatment of plaque psoriasis and psoriatic arthritis. J. Drugs Dermatol..

[13] Mocci G., Tursi A., Onidi F.M., Usai-Satta P., Pes G.M., Dore M.P. (2024). Ustekinumab in the Treatment of Inflammatory Bowel Diseases: Evolving Paradigms. J. Clin. Med..

[14] Sandborn W.J., Gasink C., Gao L.L., Blank M.A., Johanns J., Guzzo C., Sands B.E., Hanauer S.B., Targan S., Rutgeerts P. (2012). Ustekinumab induction and maintenance therapy in refractory Crohn’s disease. N. Engl. J. Med..

[15] Feagan B.G., Sandborn W.J., Gasink C., Jacobstein D., Lang Y., Friedman J.R., Blank M.A., Johanns J., Gao L.L., Miao Y. (2016). Ustekinumab as Induction and Maintenance Therapy for Crohn’s Disease. N. Engl. J. Med..

[16] Hanauer S.B., Sandborn W.J., Feagan B.G., Gasink C., Jacobstein D., Zou B., Johanns J., Adedokun O.J., Sands B.E., Rutgeerts P. (2020). IM-UNITI: Three-year Efficacy, Safety, and Immunogenicity of Ustekinumab Treatment of Crohn’s Disease. J. Crohns Colitis.

[17] Sandborn W.J., Rebuck R., Wang Y., Zou B., Adedokun O.J., Gasink C., Sands B.E., Hanauer S.B., Targan S., Ghosh S. (2022). Five-Year Efficacy and Safety of Ustekinumab Treatment in Crohn’s Disease: The IM-UNITI Trial. Clin. Gastroenterol. Hepatol..

[18] Iborra M., Beltrán B., Fernández-Clotet A., Gutiérrez A., Antolín B., Huguet J.M., De Francisco R., Merino O., Carpio D., García-López S. (2019). Real-world short-term effectiveness of ustekinumab in 305 patients with Crohn’s disease: Results from the ENEIDA registry. Aliment. Pharmacol. Ther..

[19] Liefferinckx C., Verstockt B., Gils A., Noman M., Van Kemseke C., Macken E., De Vos M., Van Moerkercke W., Rahier J.F., Bossuyt P. (2019). Long-term Clinical Effectiveness of Ustekinumab in Patients with Crohn’s Disease Who Failed Biologic Therapies: A National Cohort Study. J. Crohns Colitis.

[20] Biemans V.B.C., Van der Meulen-de Jong A.E., Van der Woude C.J., Löwenberg M., Dijkstra G., Oldenburg B., de Boer N.K.H., van der Marel S., Bodelier A.G.L., Jansen J.M. (2020). Ustekinumab for Crohn’s Disease: Results of the ICC Registry, a Nationwide Prospective Observational Cohort Study. J. Crohns Colitis.

[21] Hoffmann P., Krisam J., Wehling C., Kloeters-Plachky P., Leopold Y., Belling N., Gauss A. (2019). Ustekinumab: “Real-world” outcomes and potential predictors of non-response in treatment-refractory Crohn’s disease. World J. Gastroenterol..

[22] Eberl A., Hallinen T., Af Björkesten C.G., Heikkinen M., Hirsi E., Kellokumpu M., Koskinen I., Moilanen V., Nielsen C., Nuutinen H. (2019). Ustekinumab for Crohn’s disease: A nationwide real-life cohort study from Finland (FINUSTE). Scand. J. Gastroenterol..

[23] Bar-Gil Shitrit A., Ben-Ya’acov A., Siterman M., Waterman M., Hirsh A., Schwartz D., Zittan E., Adler Y., Koslowsky B., Avni-Biron I. (2020). Safety and effectiveness of ustekinumab for induction of remission in patients with Crohn’s disease: A multicenter Israeli study. United Eur. Gastroenterol. J..

[24] Pugliese D., Daperno M., Fiorino G., Savarino E., Mosso E., Biancone L., Testa A., Sarpi L., Cappello M., Bodini G. (2019). Real-life effectiveness of ustekinumab in inflammatory bowel disease patients with concomitant psoriasis or psoriatic arthritis: An IG-IBD study. Dig. Liver Dis..

[25] Ma C., Fedorak R.N., Kaplan G.G., Dieleman L.A., Devlin S.M., Stern N., Kroeker K.I., Seow C.H., Leung Y., Novak K.L. (2017). Clinical, endoscopic and radiographic outcomes with ustekinumab in medically-refractory Crohn’s disease: Real world experience from a multicentre cohort. Aliment. Pharmacol. Ther..

[26] Lorenzo González L., Valdés Delgado T., Vázquez Morón J.M., Laria L.C., Carnerero E.L., Maldonado Pérez M.B., Sánchez Capilla D., Pallarés Manrique H., Sáez Díaz A., Argüelles Arias F. (2022). Grupo de Enfermedad Inflamatoria de Andalucía. Ustekinumab in Crohn’s disease: Real-world outcomes and predictors of response. Rev. Esp. Enferm. Dig..

[27] Bacaksız F., Arı D., Gökbulut V., Öztürk Ö., Kayaçetin E. (2021). One-year real life data of our patients with moderate-severe Crohn’s disease who underwent ustekinumab therapy. Scott. Med. J..

[28] Macaluso F.S., Fries W., Viola A., Costantino G., Muscianisi M., Cappello M., Guida L., Giuffrida E., Magnano A., Pluchino D. (2020). Effectiveness of Ustekinumab on Crohn’s Disease Associated Spondyloarthropathy: Real-World Data from the Sicilian Network for Inflammatory Bowel Diseases (SN-IBD). Expert. Opin. Biol. Ther..

[29] Viola A., Muscianisi M., Macaluso F.S., Ventimiglia M., Cappello M., Privitera A.C., Magnano A., Pluchino D., Magrì G., Ferracane C. (2021). “Sicilian Network for Inflammatory Bowel Disease (SN-IBD)”Ustekinumab in Crohn’s disease: Real-world outcomes from the Sicilian network for inflammatory bowel diseases. JGH Open.

[30] Miranda A., Gravina A.G., Cuomo A., Mucherino C., Sgambato D., Facchiano A., Granata L., Priadko K., Pellegrino R., de Filippo F.R. (2021). Efficacy of ustekinumab in the treatment of patients with Crohn’s disease with failure to previous conventional or biologic therapy: A prospective observational real-life study. J. Physiol. Pharmacol..

[31] Yokoyama S., Asano T., Nagano K., Tsuchiya H., Takagishi M., Tsujioka S., Miura N., Matsumoto T. (2021). Safety and effectiveness of ustekinumab in Crohn’s disease: Interim results of post-marketing surveillance in Japan. J. Gastroenterol. Hepatol..

[32] Yao J.Y., Zhang M., Wang W., Peng X., Zhao J.Z., Liu T., Li Z.W., Sun H.T., Hu P., Zhi M. (2021). Ustekinumab trough concentration affects clinical and endoscopic outcomes in patients with refractory Crohn’s disease: A Chinese real-world study. BMC Gastroenterol..

[33] Tursi A., Mocci G., Cuomo A., Allegretta L., Aragona G., Colucci R., Della Valle N., Ferronato A., Forti G., Gaiani F. (2021). Real-life efficacy and safety of ustekinumab as second- or third-line therapy in Crohn’s disease: Results from a large Italian cohort study. Eur. Rev. Med. Pharmacol. Sci..

[34] Scribano M.L., Aratari A., Neri B., Bezzio C., Balestrieri P., Baccolini V., Falasco G., Camastra C., Pantanella P., Monterubbianesi R. (2022). Effectiveness of ustekinumab in patients with refractory Crohn’s disease: A multicentre real-life study in Italy. Therap Adv. Gastroenterol..

[35] Ylisaukko-Oja T., Puttonen M., Jokelainen J., Koivusalo M., Tamminen K., Torvinen S., Voutilainen M. (2022). Dose-escalation of adalimumab, golimumab or ustekinumab in inflammatory bowel diseases: Characterisation and implications in real-life clinical practice. Scand. J. Gastroenterol..

[36] Bennett A., Carlini L.E., Duley C., Garrett A., Annis K., Wagnon J., Dalal R., Scoville E., Beaulieu D., Schwartz D. (2020). A Single Center Experience with Long-Term Ustekinumab Use and Reinduction in Patients with Refractory Crohn Disease. Crohns Colitis 360.

[37] Parra R.S., Chebli J.M.F., Queiroz N.S.F., Damião A.O.M.C., de Azevedo M.F.C., Chebli L.A., Bertges E.R., Alves Junior A.J.T., Ambrogini Junior O., da Silva B.L.P.S. (2022). Long-term effectiveness and safety of ustekinumab in bio-naïve and bio-experienced anti-tumor necrosis factor patients with Crohn’s disease: A real-world multicenter Brazilian study. BMC Gastroenterol..

[38] Forss A., Clements M., Myrelid P., Strid H., Söderman C., Wagner A., Andersson D., Hjelm F., Olén O., PROSE SWIBREG study group (2023). Ustekinumab Is Associated with Real-World Long-Term Effectiveness and Improved Health-Related Quality of Life in Crohn’s Disease. Dig. Dis. Sci..

[39] Esaki M., Ihara Y., Tominaga N., Takedomi H., Tsuruoka N., Akutagawa T., Yukimoto T., Kawasaki K., Umeno J., Torisu T. (2023). Predictive factors of the clinical efficacy of ustekinumab in patients with refractory Crohn’s disease: Tertiary centers experience in Japan. Int. J. Colorectal Dis..

[40] Casas-Deza D., Lamuela-Calvo L.J., Gomollón F., Arbonés-Mainar J.M., Caballol B., Gisbert J.P., Rivero M., Sánchez-Rodríguez E., Arias García L., Gutiérrez Casbas A. (2023). Effectiveness and Safety of Ustekinumab in Elderly Patients with Crohn’s Disease: Real World Evidence From the ENEIDA Registry. J. Crohns Colitis.

[41] Wils P., Bouhnik Y., Michetti P., Flourie B., Brixi H., Bourrier A., Allez M., Duclos B., Serrero M., Buisson A. (2018). Groupe d’Etude Therapeutique des Affections Inflammatoires du Tube Digestif (GETAID). Long-term efficacy and safety of ustekinumab in 122 refractory Crohn’s disease patients: A multicentre experience. Aliment. Pharmacol. Ther..

[42] Ma C., Fedorak R.N., Kaplan G.G., Dieleman L.A., Devlin S.M., Stern N., Kroeker K.I., Seow C.H., Leung Y., Novak K.L. (2017). Long-term maintenance of clinical, endoscopic, and radiographic response to Ustekinumab in moderate-to-severe Crohn’s disease: Realworld experience from a multicenter cohort study. Inflamm. Bowel Dis..

[43] Chebli J.M.F., Parra R.S., Flores C., Moraes A.C., Nones R.B., Gomes T.N.F., Perdomo A.M.B., Scapini G., Zaltman C. (2022). Effectiveness and safety of Ustekinumab for moderate to severely active Crohn’s disease: Results from an early access program in Brazil. J. Clin. Med..

[44] Regime di Rimborsabilità e Prezzo, a Seguito di Nuove Indicazioni Terapeutiche, del Medicinale per uso Umano “Stelara”. Gazzetta Ufficiale della Repubblica Italiana del 03.09.2018; Serie Generale-n. 204: 15-18. https://www.gazzettaufficiale.it/atto/serie_generale/caricaDettaglioAtto/originario?atto.dataPubblicazioneGazzetta=2018-09-03&atto.codiceRedazionale=18A05714&elenco30giorni=false.

[45] Gomollón F., Dignass A., Annese V., Tilg H., Van Assche G., Lindsay J.O., Peyrin-Biroulet L., Cullen G.J., Daperno M., Kucharzik T. (2017). 3rd European Evidence-based Consensus on the Diagnosis and Management of Crohn’s Disease 2016: Part 1: Diagnosis and Medical Management. J Crohns Colitis.

[46] Lamb C.A., Kennedy N.A., Raine T., Hendy P.A., Smith P.J., Limdi J.K., Hayee B., Lomer M.C.E., Parkes G.C., Selinger C. (2019). Britich Society of Gastroenterology Consensus Guidelines on the management of Inflammatory Bowel Diseases in Adults. Gut.

[47] Satsangi J., Silverberg M.S., Vermeire S., Colombel J.F. (2006). The Montreal classification of inflammatory bowel disease: Controversies, consensus, and implications. Gut.

[48] Best W.R. (2006). Predicting the Crohn’s Disease activity index from the Harvey–Bradshaw Index. Inflamm. Bowel Dis..

[49] Daperno M., D’Haens G., Van Assche G., Baert F., Bulois P., Maunoury V., Sostegni R., Rocca R., Pera A., Gevers A. (2004). Development and validation of a new, simplified endoscopic activity score for Crohn’s disease: The SES-CD. Gastrointest. Endosc..

[50] Moskovitz D.N., Daperno M., Van Assche G. (2007). Defining and validating cut-offs for the simple endoscopic score for Crohn’s disease. Gastroenterology.

[51] Rutgeerts P., Geboes K., Vantrappen G., Vantrappen G., Beyls J., Kerremans R., Hiele M. (1990). Predictability of the postoperative course of Crohn’s disease. Gastroenterology.

[52] Colombel J.F., Sandborn W.J., Reinisch W., Mantzaris G.J., Kornbluth A., Rachmilewitz D., Lichtiger S., D’Haens G., Diamond R.H., Broussard D.L. (2010). SONIC Study Group. Infliximab, azathioprine, or combination therapy for Crohn’s disease. N. Engl. J. Med..

[53] (2016). Stelara (Ustekinumab) [Package Insert], in U.S. Food and Drug Administration website. Janssen Pharmaceutical Companies. https://www.accessdata.fda.gov/drugsatfda_docs/label/2016/761044lbl.pdf.

[54] Macaluso F.S., Maida M., Ventimiglia M., Ventimiglia M., Cottone M., Orlando A. (2020). Effectiveness and safety of Ustekinumab for the treatment of Crohn’s disease in real-life experiences: A meta-analysis of observational studies. Expert. Opin. Biol. Ther..

[55] (2023). Ustekinumab reintroduction: Week 16 results and baseline response analysis from the POWER study in patients with Crohn’s disease. Gastroenterol. Hepatol..

[56] Schmitt H., Billmeier U., Dieterich W., Rath T., Sonnewald S., Reid S., Hirshmann S., Hildner K., Waldner M.J., Mudter J. (2019). Expansion of IL-23 receptor bearing TNFR2+ T cells is associated with molecular resistance to anti-TNF therapy in Crohn’s disease. Gut.

[57] Yang J.Y., Lund J.L., Funk M.J., Hudgens M.G., Lewis J.D., Kappelman M.D., SPARC IBD Investigators (2023). Utilization of Treat-to-Target Monitoring Colonoscopy After Treatment Initiation in the US-Based Study of a Prospective Adult Research Cohort With Inflammatory Bowel Disease. Am. J. Gastroenterol..

[58] Alizadeh M., Ali O., Cross R.K. (2024). Assessing Progression of Biologic Therapies Based on Smoking Status in Patients With Crohn’s Disease. Inflamm. Bowel Dis..

